# Reorientation of the diagonal double-stripe spin structure at Fe_1+*y*_Te bulk and thin-film surfaces

**DOI:** 10.1038/ncomms13939

**Published:** 2017-01-06

**Authors:** Torben Hänke, Udai Raj Singh, Lasse Cornils, Sujit Manna, Anand Kamlapure, Martin Bremholm, Ellen Marie Jensen Hedegaard, Bo Brummerstedt Iversen, Philip Hofmann, Jin Hu, Zhiqiang Mao, Jens Wiebe, Roland Wiesendanger

**Affiliations:** 1 Department of Physics, Hamburg University, Jungiusstrasse 9A, 20355 Hamburg, Germany; 2 Center for Materials Crystallography, Department of Chemistry and iNANO, Aarhus University, DK-8000 Aarhus C, Denmark; 3 Department of Physics and Astronomy, Interdisciplinary Nanoscience Center, Aarhus University, DK-8000 Aarhus C, Denmark; 4 Department of Physics and Engineering Physics, Tulane University, New Orleans, Los Angeles 70118, USA

## Abstract

Establishing the relation between ubiquitous antiferromagnetism in the parent compounds of unconventional superconductors and their superconducting phase is important for understanding the complex physics in these materials. Going from bulk systems to thin films additionally affects their phase diagram. For Fe_1+*y*_Te, the parent compound of Fe_1+*y*_Se_1−*x*_Te_*x*_ superconductors, bulk-sensitive neutron diffraction revealed an in-plane oriented diagonal double-stripe antiferromagnetic spin structure. Here we show by spin-resolved scanning tunnelling microscopy that the spin direction at the surfaces of bulk Fe_1+*y*_Te and thin films grown on the topological insulator Bi_2_Te_3_ is canted out of the high-symmetry directions of the surface unit cell resulting in a perpendicular spin component, keeping the diagonal double-stripe order. As the magnetism of the Fe *d*-orbitals is intertwined with the superconducting pairing in Fe-based materials, our results imply that the superconducting properties at the surface of the related superconducting compounds might be different from the bulk.

## Abstract (web summary)

Knowledge of the spin structure in parent compounds of unconventional superconductors is crucial for an understanding of the complex physics in these materials. Here, the authors report canted spin structure on the surface as well as on the thin film form of Fe_1+*y*_Te, different from the bulk.

The physics of many transition metal oxides (TMOs) is dominated by strong electronic correlations, which leads to exotic ground states and excitations with a dominant role of electronic charge and spin degrees of freedom. For example, for cuprate-based high-temperature superconductors there have been growing evidence that charge and spin density wave (SDW)-like states are inherent to these materials with a significant impact on the corresponding excitation spectrum^1^,^2^,^3^. Indeed, spin and charge ordering appear to be a key feature for understanding the physics of high-temperature superconductors^4^,^5^. Furthermore, among the correlated electron systems the recently discovered iron-based superconductors^6^ are of particular interest for the understanding of the interplay between superconductivity and magnetism. Especially, the iron-chalcogenide system Fe_1+*y*_Se_1−*x*_Te_*x*_ has gained high interest due to the surprisingly high *T*_C_ superconductivity in single-layer films of FeSe^7^,^8^,^9^ and its unique interplay between magnetism and superconductivity^10^. For bulk single crystals, FeSe exhibits a superconducting transition temperature *T*_C_ of no higher than 10 K (ref. 11) but in a single-unit cell (UC) thick film grown on SrTiO_3_ *T*_C_ can be increased above 100 K (ref. 9). This finding has spurred numerous investigations aiming at the understanding of how superconductivity evolves in transition metal chalcogenides from bulk to ultra-thin films. In particular, scanning tunnelling microscopy (STM) with atomic-scale resolution has proven to be an indispensable tool for revealing the real-space electronic structure^12^,^13^. However, most of the recent STM studies on TMO and iron-based superconductors have been focusing only on the charge degrees of freedom. With the recent developments in spin-polarized STM (SP-STM)^14^,^15^, which accesses both charge and spin degrees of freedom on the atomic length scale, detailed investigations of TMO compounds and iron-based superconductors have now become possible.

As Fe_1+*y*_Te exhibits diagonal double-stripe antiferromagnetic (DDS) spin order, contrasted with single-stripe antiferromagnetic (AFM) order in iron pnictide superconductor parent compounds, the study on its mechanism of magnetic order has recently attracted a lot of attention. It provides a non-polar charge-neutral Te-terminated surface on cleaving^16^,^17^,^18^. Furthermore Fe_1+*y*_Te can be grown *in situ* by molecular beam epitaxy with high quality^19^,^20^. In bulk, Fe_1+*y*_Te is the non-superconducting parent compound of the transition metal chalcogenide system Fe_1+*y*_Se_1−*x*_Te_*x*_^10^ and exhibits the DDS magnetic structure below its Néel-temperature *T*_N_^21^,^22^,^23^. Depending on its excess iron concentration *y*, the Néel temperature varies from *T*_N_≈60–70 K^24^. Using neutron diffraction, it has been shown that in the bulk the Fe spins within the DDS structure are pointing along the diagonal of the Fe–Fe square network^22^,^23^. The crystal structure and corresponding DDS order are schematically shown in Fig. 1a,b. The magnetic phase transition of bulk Fe_1+*y*_Te is accompanied by a structural phase transition, with the structure changing from a tetragonal to a monoclinic phase for which the lattice constant *a*_Te_ is slightly larger than the lattice constant *b*_Te_^22^,^25^. The DDS structure itself shows a commensurate AFM modulation along the *a*_Te_ direction with a wave length of *λ*_AFM_=2*a*_Te_ and has a ferromagnetic coupling along the *b*_Te_ direction^22^,^23^,^26^.

STM data obtained for Fe_1+*y*_Te bulk and thin film samples have revealed atomic resolution of the Te-terminated surface and a superstructure on top of the atomic corrugation having a periodicity of *λ*=2*a*_Te_^18^,^19^,^20^,^27^,^28^,^29^. The observation of this additional *λ*=2*a*_Te_ periodicity is mainly discussed in two contradictory models. On one hand, the interpretation for the 2*a*_Te_ superstructure is given in terms of a charge density wave (CDW), where the charge density of the top Te layer is modified by the SDW of the underlying AFM order of the Fe layer. The strong dependence on the bias voltage *V*_bias_ suggests a complex interplay between the charge and the magnetic order^18^,^20^,^27^,^30^. In contrast to a common representation of spin and charge modulations in correlated electron systems^31^, in this case the CDW would have the same periodicity as the SDW. Opposed to this interpretation, experiments performed with magnetically sensitive tips, which were prepared by attaching excess Fe atoms to the tip apex, give additional insight based on spin-polarized tunnelling^28^,^29^. The 2*a*_Te_ superstructure can unambiguously be assigned to a direct imaging of the DDS order of the underlying Fe lattice. However, in previous studies the absolute orientation of the spins within the AFM structure could not be revealed.

In this work we investigated the Fe_1+*y*_Te surface of bulk samples and of thin Fe_1+*y*_Te films grown on Bi_2_Te_3_ by SP-STM, revealing the DDS structure in both sample systems. Moreover, by using Fe-coated W-tips in a vector-magnet system, we were able to rotate the tip magnetization direction both within the surface plane as well as perpendicular to the surface. As we thereby have access to the different components of the spin direction of the sample, we could unambiguously prove that the spin direction within the surface DDS structure is canted with respect to the crystallographic *b*_Te_ direction.

## Results

### SP-STM on the surface of Fe_1+*y*_Te bulk and thin films

In spin-polarized STM, the tunnelling current depends on the relative orientation of the tip magnetization and the local spin direction of the sample. The total tunnelling current *I*_t_ can be described by *I*_t_=*I*_0+_*I*_p_, where *I*_0_ is the spin-averaged tunnelling current and *I*_p_ is the spin-polarized tunnelling current, which is proportional to the product of the spin polarization *P*_t_ of tip and the energy integrated spin polarization *P*_s_ of the sample, *I*_p_∝*P*_t_·*P*_s_·cos(*β*). Here, *β* is the angle between the spin directions of the tip and the sample^32^.

In general, Fe-coated W-tips have a magnetization direction perpendicular to the tip axis and thus parallel to the surface of the sample. Therefore, Fe-coated W-tips exhibit sensitivity to an a priori unknown in-plane component of the sample magnetization. By applying an external magnetic field of about 1 T, the magnetization direction of the tip will be reoriented parallel to the applied field direction^33^. However, the corresponding Zeeman energy of 0.1 meV is too weak to affect the spin structure of Fe_1+*y*_Te, as the exchange interactions between neighbouring Fe atoms in the DDS structure are on the order of 10 meV (ref. 34), and there is a considerable magnetic anisotropy energy of about 0.5 meV per Fe atom^28^. By application of the external magnetic field in different orientations and recording constant-current SP-STM images, we can therefore image the particular component of the sample spin structure in the given direction of the magnetic field.

**Figure 1:**
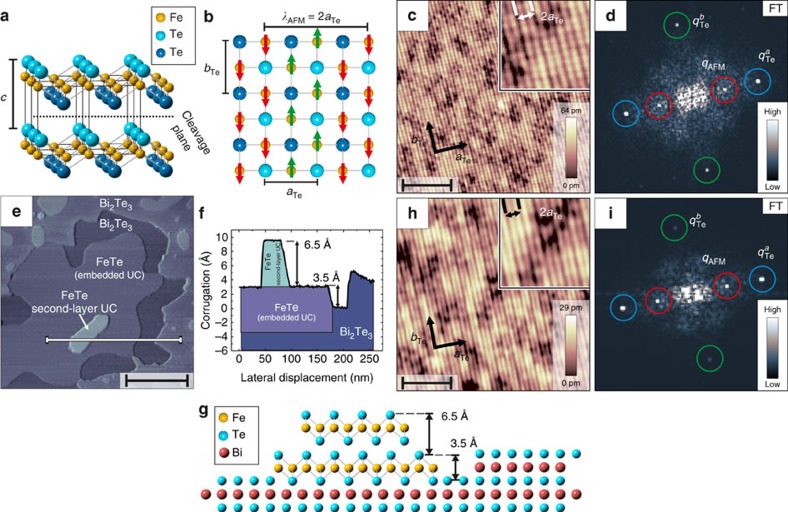
Structure and magnetic contrast of the investigated Fe_1+*y*_Te bulk and thin film samples. (**a**) Crystal structure of bulk Fe_1+*y*_Te showing four UCs. (**b**) Top view of the Te terminated surface and the underlying Fe lattice. The spin direction of the bulk DDS order is indicated by the red and green arrows (the image is adapted from refs 22, 23). (**c**) SP-STM image of the surface of Fe_1+*y*_Te measured with an Fe-coated W-tip showing the atomic and the spin structure (*V*_bias_=+50 mV; *I*_t_=320 pA; *B*=0 T; scale bar, 5 nm). White arrows denote the lattice directions *a*_Te_ and *b*_Te_. Inset: magnified image showing the atomic lattice of the surface Te atoms with a 2*a*_Te_ periodic superstructure. (**d**) The FT of **c**, indicating the Bragg peaks 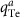 (blue circles), the Bragg peaks 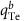 (green circles) and the peaks *q*_AFM_ of the 2*a*_Te_ magnetic superstructure (red circles). (**e**) Topographic overview of the second-layer island growth of Fe_1+*y*_Te on Bi_2_Te_3_ (scale bar, 120 nm). (**f**) Plotted line section along the white line shown in **e**. (**g**) Model of the investigated morphology of Fe_1+*y*_Te grown on Bi_2_Te_3_ along the section in **f**. (**h**) SP-STM image of the two UC thin layer of Fe_1+*y*_Te grown on Bi_2_Te_3_ measured with an Fe-coated W-tip showing the atomic structure together with an additional 2*a*_Te_ spin contrast (*V*_bias_=+33 mV; *I*_t_=4.1 nA; *B*=2.5 T out-of-plane; scale bar, 3.8 nm). It is noteworthy that for this particular data the tips' spin polarization is perpendicular to the sample spin orientation. Therefore, a non-zero magnetic field had to be used to rotate the tip magnetization, to observe the 2*a*_Te_ spin contrast. White arrows denote the lattice directions *a*_Te_ and *b*_Te_. Inset: magnified image showing the atomic lattice of the surface Te atoms with a 2*a*_Te_ periodic superstructure. (**i**) The FT of **h** with the same assignment of the different peaks as in **d**.

In Fig. 1, we give an introduction to both Fe_1+*y*_Te samples used in this work. The first investigated sample shown in Fig. 1c,d is the surface of cleaved bulk Fe_1+*y*_Te (*y*∼0.07). Figure 1c displays a typical spin-resolved constant-current image of the bulk Fe_1+*y*_Te surface, which shows clear atomic resolution of the Te-terminated surface (a detailed analysis of the investigated spin contrast is discussed later on). Compared with previously reported results^18^,^28^,^30^, a large area of the Fe_1+*y*_Te surface is free of excess Fe atoms, which is due to the annealing procedure as described in the Methods section. This annealing procedure leads to the formation of Fe clusters containing all the excess Fe (outside of the field of view of Fig. 1c) and large areas with no surface excess Fe atoms in between these clusters. The surface is atomically flat but shows a small variation in the apparent height, which is probably caused by excess Fe atoms between the sub-surface layers. By calculating the Fourier transform (FT) of Fig. 1c, the lattice periodicity is displayed as bright Bragg spots labelled as 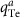 and 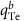 in Fig. 1d, where 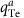 has a higher intensity than 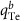 (*cf*. refs 20, 28, 30). In addition to the atomic periodicity the constant-current image in Fig. 1c shows the typical superstructure, which has a periodicity of *λ*=2*a*_Te_ along the *a*_Te_ direction. The presence of this superstructure is visible as additional spots in the FT (labelled with *q*_AFM_) with a wave vector of *q*_AFM_=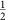

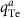
. Overall, the cleaved surface shows all the characteristics previously reported for Fe_1+*y*_Te (*cf*. refs 20, 27, 28).

The second investigated sample is a thin Fe_1+*y*_Te film grown on Bi_2_Te_3_ (Fig. 1e–g). A large-scale STM topography of the sample area is displayed in Fig. 1e together with the height profile along a line shown in Fig. 1f. From atomically resolved images taken on the different visible layers and their apparent heights, we deduce the structure of the layers as sketched in Fig. 1g. The first UC thin layer of FeTe, which has an apparent hight of ∼3.5 Å (Fig. 1e), is embedded into an incomplete Bi_2_Te_3_ quintuple layer. Owing to the interaction with the underlying substrate it exhibits a network of stripe-like dislocations, leading to a rough surface (not resolved in the large scale image of Fig. 1e). On top of the embedded layer, the growth of an additional FeTe layer has started and forms a second layer island with a height of 6.5 Å, which is roughly equal to the *c* axis lattice constant (6.26 Å)^23^ of the bulk Fe_1+*y*_Te UC as shown in Fig. 1a. This second layer has an atomically flat surface. In this work we focused on SP-STM measurements on top of the second layer islands indicated by the white arrow in Fig. 1e. In Fig. 1h, an atomically resolved image of the surface on the second layer Fe_1+*y*_Te island is shown. We do not observe excess Fe atoms on the surface, but we cannot exclude that there is a small amount of excess Fe atoms sitting in the van der Waals gap in between the two FeTe layers. In addition to the atomic corrugation the characteristic 2*a*_Te_ superstructure is visible as indicated in the inset of Fig. 1h, which is very similar to the one at the bulk Fe_1+*y*_Te surface shown in Fig. 1c. This is also visible in the FT displayed in Fig. 1i, which coincides with the FT pattern in Fig. 1d; the atomic lattice shows peaks of different intensity (labelled with 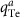 and 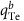) and also the 2*a*_Te_ superstructure peaks are visible (labelled with *q*_AFM_). Therefore, the spin-resolved STM image of the second layer Fe_1+*y*_Te island exhibits a very similar spin structure as the surface of bulk Fe_1+*y*_Te. It is noteworthy that for both the bulk samples and the thin film samples, the 2*a*_Te_ superstructure is observed only if the tip is magnetically coated, or if a nominally uncoated tip picks up surface Fe atoms or a cluster of FeTe due to a tip-sample contact, as also shown in ref. 28. Images taken with a zero spin polarization at the foremost atom of the tip show only the atomic contrast from the topmost Te atoms with a periodicity of *a*_Te_ and *b*_Te_ (Supplementary Fig. 1).

### Magnetic field-dependent SP-STM on bulk Fe_1+*y*_Te

**Figure 2:**
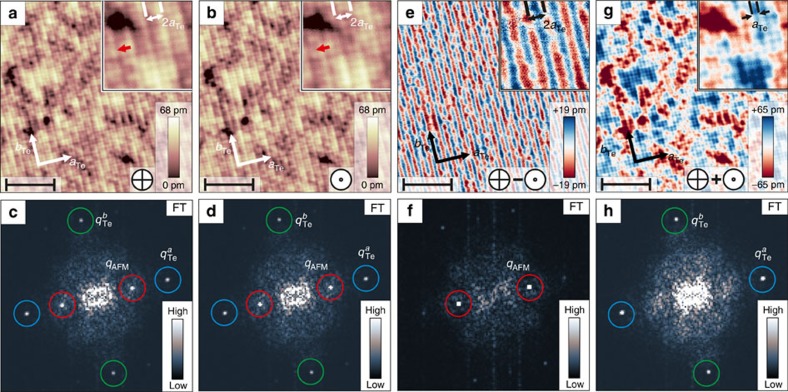
SP-STM images revealing the DDS order at the surface of bulk Fe_1+*y*_Te. For the constant-current images of (**a**,**b**) (*V*_bias_=+50 mV and *I*_t_=340 pA) an out-of-plane external magnetic field of *B*=±1 T was applied. The direction of the external magnetic field is indicated by the arrows pointing into or out of the surface plane. White arrows denote the lattice directions *a*_Te_ and *b*_Te_. Insets: magnified images showing the atomic lattice of the surface Te atoms with a 2*a*_Te_ periodic superstructure. The red arrow denotes the same location on the sample, where due to the opposite direction of the tip magnetization in **a**, a minimum of the 2*a*_Te_ modulation is observed, and in **b** a maximum. (**c**,**d**) The FTs of the constant-current maps in **a**,**b**. (**e**) Difference image of **a**,**b** consistent with a DDS spin structure of the Fe_1+*y*_Te surface. Inset: magnified image showing the 2*a*_Te_ periodic superstructure. (**f**) The corresponding FT of **e**. (**g**) Sum image of **a**,**b**. Inset: magnified image showing only the atomic lattice of the surface Te atoms without the 2*a*_Te_ periodic superstructure. (**h**) The corresponding FT of **g**. Scale bars, 5 nm wide (**a**,**b**,**e**,**g**). The peaks in the FTs (**c**,**d**,**f**,**h**) are labelled with blue circles (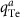), green circles (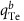) and red circles (*q*_AFM_).

After introducing the two different sample systems used in this work we will now discuss our results obtained with different orientations of the tip magnetization starting with the surface of bulk Fe_1+*y*_Te and out-of-plane tip sensitivity. Figure 2a,b show constant-current maps of the same surface area obtained with an Fe-coated tip. They were recorded in a magnetic field of 1 T with opposite out-of-plane field directions, which forces the tip magnetization to point up and down. The two SP-STM images show the characteristic 2*a*_Te_ superstructure, which is also visible by the *q*_AFM_ peak displayed in the corresponding FTs in Fig. 2c,d. In contrast to different interpretations such as charge ordering phenomena^18^,^20^, this additional superstructure has been attributed to a direct imaging of the DDS order of Fe_1+*y*_Te by SP-STM^28^,^29^. Our experiments show that this is indeed the case and confirm that spin-polarized tunnelling is the origin of the additional 2*a*_Te_ superstructure by applying external magnetic fields. By comparing the superstructure with the atomic lattice, a phase shift by one lattice unit is observed for opposite magnetic field directions (*cf*. insets of Fig. 2a,b). By subtracting the constant-current maps in Fig. 2a from that in Fig. 2b, an image of the out-of-plane components of the spin structure is obtained in Fig. 2e, which mainly shows a stripe pattern with a periodicity of 2*a*_Te_ along the *a*_Te_ direction. This is also reflected by the FT of the difference image in Fig. 1f. Here, only the peak *q*_AFM_ remains and the Bragg peaks 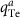 and 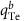 related to the atomic lattice have vanished. On the other hand, the sum image of the constant-current maps in Fig. 2a,b, which is shown in Fig. 2g together with its FT in Fig. 2h, does not reveal the 2*a*_Te_ periodic stripe pattern, but merely the Bragg peaks 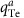 and 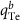. This observation directly confirms the results from ref. 28 and proves spin-polarized tunnelling contrast due to the DDS spin structure of Fe_1+*y*_Te. The maximum spin contrast appears between every second Fe lattice site located between two neighbouring Te sites. This can be explained by the fact that spin-polarized tunnelling primarily results from the 3*d* states of Fe being located below the top Te layer.

However, from the strong spin contrast we see using the out-of-plane sensitive magnetic tip, we can additionally conclude that the surface AFM structure has a considerable out-of-plane spin component. Considering the magnetic structure of bulk Fe_1+*y*_Te as known from neutron diffraction^21^,^22^,^23^ depicted in Fig. 1b, this leads to the conclusion that the surface spins are reoriented with respect to the corresponding bulk layers.

**Figure 3:**
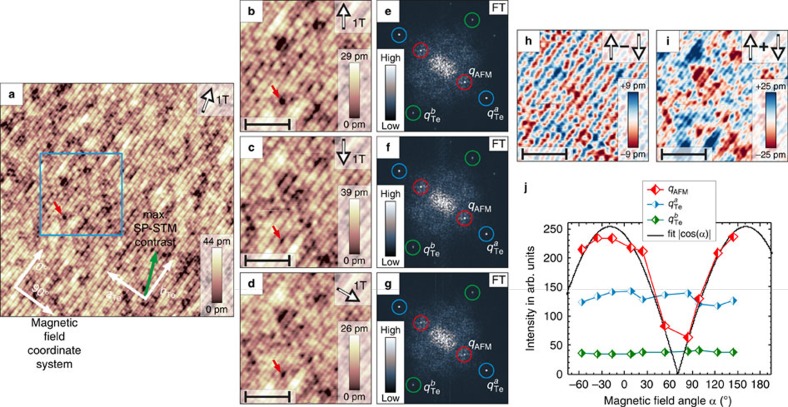
In-plane tip-magnetization direction-dependent spin contrast at the surface of bulk Fe_1+*y*_Te. (**a**) Spin-resolved (32.5 × 32.5) nm^2^ overview measured in an in-plane external magnetic field of 1 T at −16° (*V*_bias_=+50 mV and *I*_t_=500 pA). (**b**,**c**) The magnetic contrast at opposite field directions at −36° and 144° (|*B*|=1 T) revealing a phase shift, which is shown in the difference image in **h**. (**d**) Almost vanishing magnetic contrast at an angle of 84° (|*B*|=1 T). The red arrows in **b**–**d** indicate an atomic scale defect, that is, point at the identical positions in all images. In all SP-STM images, the direction of the applied field is indicated by the arrows in the insets. (**e**–**g**) The FTs of **b**–**d**, respectively. The spots in the FTs are labelled with blue circles (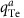), green circles (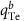) and red circles (*q*_AFM_). (**i**) The sum image of **b**,**c**. Scale bars 3.8 nm wide (**b**–**d**,**h**,**i**). (**j**) The plotted intensity of the three components 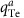, 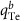 and *q*_AFM_ of the FTs. The colour coding of the corresponding symbols match the circular markings in the FTs.

To analyse the in-plane surface spin components of the DDS structure of bulk Fe_1+*y*_Te, we performed experiments within a vector-magnet system, which enables to rotate the tip magnetization direction within the film plane. For this purpose a magnetic field with a fixed amplitude was applied parallel to the surface and then stepwise rotated for each taken SP-STM image recorded. The results are shown in Fig. 3 for the magnetic field amplitude of 1 T. Figure 3a shows an overview of the atomically resolved Fe_1+*y*_Te surface with a magnetic field applied under an angle of *α*=−16° relative to the given magnetic field coordinate system. On top of the atomic corrugation, the strong 2*a*_Te_ superstructure is visible running continuously through the whole image from the bottom left to the top right. The blue square indicates the area where the investigations with rotated magnetic fields were performed and the red arrow indicates a defect used as a marker for the atomic-scale registry. On rotating the magnetic field parallel to the surface, the intensity of the 2*a*_Te_ superstructure was measured by taking spin-resolved constant-current maps for each field direction (Fig. 3b–d, see full set of images in Supplementary Fig. 2). The intensity of the superstructure was then extracted from the amplitude of the *q*_AFM_ peak in the FTs (Fig. 3e–g). In addition to the *q*_AFM_ peak, the intensity of the Bragg peaks 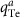 and 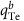 were recorded as a reference, to confirm that the tip did not change during the full magnetic field sweep. These intensities are shown in Fig. 3j as a function of the magnetic field angle. In Fig. 3b,c, the tip magnetization direction is pointing in opposite directions within the surface plane. It is again apparent that the 2*a*_Te_ superstructure observed for both field directions exhibits a phase shift of one lattice unit and is merely a result of the spin-sensitivity of the tip. This is verified by calculating the difference and sum of Fig. 3b,c shown in Fig. 3h,i, where only the magnetic signal 2*a*_Te_ of the superstructure, or the purely electronic signal with the lattice periodicity, respectively, remain. By comparing the intensities of the two Bragg peaks 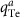 and 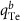, and the DDS *q*_AFM_ peak extracted from the FTs of these images shown in Fig. 3e,f, we can conclude that both SP-STM images exhibit the same amplitudes for the atomic corrugation and the 2*a*_Te_ superstructure, respectively, as shown in the plot of Fig. 3j. In contrast, in the SP-STM image and its FT for a field direction of 84° (Fig. 3d,g), the amplitude of the atomic corrugation remains at the same level but the amplitude of the 2*a*_Te_ superstructure is strongly reduced. Overall, the resulting angular dependence plotted in Fig. 3j reveals a periodic variation of the *q*_AFM_ peak intensity with a periodicity of 180°, whereas the intensities of the Bragg peaks do not show significant changes. As shown by the fitted line, the *q*_AFM_ intensity nicely follows a |cos(*α*)|-dependence, which is expected for spin-polarized tunnelling into an AFM spin structure on the rotation of the tip magnetization. The maximum of the intensity was found to be at −19°. The corresponding in-plane spin direction of the DDS structure is indicated by the green arrow in Fig. 3a. We can thus deduce that the DDS spin structure of the surface layer has an in-plane component, which deviates by 19° from the *b*_Te_ direction, the spin direction of the DDS spin structure in the bulk layers of Fe_1+*y*_Te. Moreover, considering the similar *q*_AFM_ intensity for the out-of-plane case (Fig. 2c,d) and for the maximum in-plane contrast case (Fig. 3j), we can conclude that the DDS spin structure at the surface is additionally rotated out of the surface plane by roughly 45°.

### Magnetic field-dependent SP-STM on Fe_1+*y*_Te films on Bi_2_Te_3_

**Figure 4:**
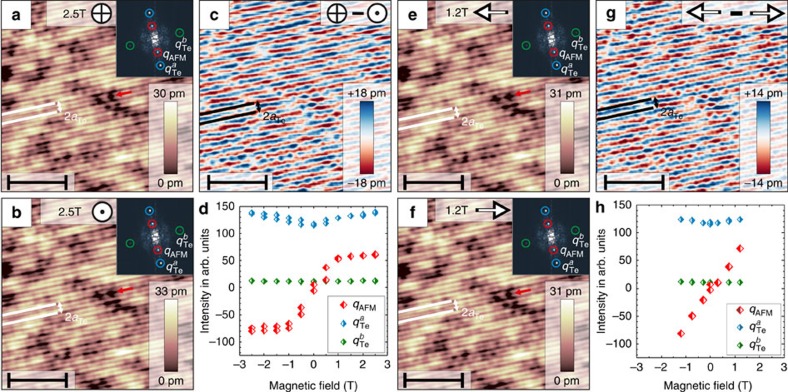
SP-STM revealing the DDS spin structure at the surface of Fe_1+*y*_Te thin films grown on Bi_2_Te_3_. (**a**,**b**,**e**,**f**) The magnetic contrast in the same area of a two-layer thick Fe_1+*y*_Te island measured with the same tip where in **a**,**b** ±2.5 T were applied in the out-of-plane direction, whereas in **e**,**f** ±1.2 T were applied in the in-plane direction (*V*_bias_=+33 mV and *I*_t_=4.1 nA). The red arrows indicate a defect used as a marker. The direction of the applied magnetic field is indicated by the arrows. The insets in the upper right of **a**,**b**,**e**,**f** display the FTs of each SP-STM image and the spots in the FTs are labelled with blue circles (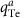), green circles (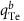) and red circles (*q*_AFM_). (**c**) The different image of **a**,**b**. (**g**) The difference image of **e**,**f**. Scale bars 5.8 nm wide (**a**–**c**,**e**–**g**). The magnetic field dependence of the intensity of 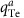, 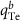 and *q*_AFM_ in the FTs for out-of-plane magnetic fields is plotted in **d** and for magnetic fields applied in the in-plane direction in **h**. The colour coding of the corresponding symbols match the circular markings in the FTs. Since the absolute value of the FT contains no phase information the amplitude of the *q*_AFM_ peak was multiplied by −1 for negative field directions to illustrate the observed phase shift of the DDS superstructure.

For comparison, we will now discuss the results obtained for a thin Fe_1+*y*_Te film grown on a Bi_2_Te_3_ substrate. All SP-STM data in Fig. 4 were obtained in the same surface area on the top of a two UC high Fe_1+*y*_Te island for magnetic fields of 2.5 T applied in opposite out-of-plane directions (Fig. 4a,b) and for magnetic fields of 1.2 T applied in opposite in-plane directions (Fig. 4e,f). As for the measurements discussed above, the characteristic 2*a*_Te_ superstructure is observed in all four SP-STM data sets. The FTs in the insets of Fig. 4a,b,e,f also show the same *q*_AFM_ pattern as for the bulk samples. By comparing the position of the maximum of the 2*a*_Te_ superstructure relative to the underlying Te lattice, for example, at the position indicated by the red arrow, the Fe_1+*y*_Te thin-film system also reveals a phase shift by one lattice constant upon inverting of the tip's magnetization direction in the out-of-plane and in the in-plane direction. This is also obvious from the difference images in Fig. 4c,g, reflecting the images of the out-of-plane and in-plane components of the spin structure, respectively. Therefore, we can conclude that the surface of the thin film Fe_1+*y*_Te on Bi_2_Te_3_ exhibits the same DDS spin structure as the bulk samples. In addition, to analyse the direction of the surface spins, the magnetic field dependence of the intensities of the *q*_AFM_ peak and of the Bragg peaks 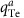 and 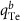 were extracted for the out-of-plane and for the in-plane direction and are shown in Fig. 4d,h, respectively (full series of magnetic field dependent SP-STM images is given in the Supplementary Figs 3 and 4). For zero magnetic field, the 2*a*_Te_ superstructure has vanished and the intensity of the *q*_AFM_ peak is basically zero. For this particular tip, the initial tip magnetization at zero magnetic field is thus almost perpendicular to the spin direction in the Fe_1+*y*_Te surface layer. It is noteworthy that the absence of the 2*a*_Te_ superstructure for this case again implies that there is no contribution to the superstructure from a CDW, in conflict with previous results^18^,^20^,^27^. On increasing the magnetic field in the out-of-plane direction the intensity of *q*_AFM_ starts changing, as the magnetization direction of the tip is continuously rotated from the in-plane to the out-of-plane direction as shown in Fig. 4d. For both the negative and the positive field direction, the intensity of *q*_AFM_ saturates when the tip magnetization is fully rotated into the out-of-plane direction. For the in-plane direction a similar behaviour is observed as shown in Fig. 4h. On increasing the magnetic field, the tip continuously rotates into the direction of the magnetic field, where a strong magnetic contrast is deduced by the increase in the *q*_AFM_ intensity. From the similar saturation values of the *q*_AFM_ intensities in Fig. 4d,h, we can conclude that the out-of-plane component of the DDS spin structure in the surface layer of the Fe_1+*y*_Te thin film has a similar strength as the in-plane component, that is, the spin structure is rotated by roughly 45° out of the surface plane.

## Discussion

In summary, we provide a direct proof that spin-polarized tunnelling is responsible for the observation of the 2*a*_Te_ superstructure in STM on Fe_1+*y*_Te by using well-defined spin-sensitive Fe-coated W-tips. Measurements under applied magnetic fields reveal that the 2*a*_Te_ superstructure can only be interpreted in terms of direct SP-STM imaging of the DDS order of the Fe_1+*y*_Te surface. This confirms previous SP-STM results with magnetically sensitive tips on Fe_1+*y*_Te^28^,^29^ and does not support the interpretation of a 2*a*_Te_ CDW-order discussed in refs 18, 20, 27. Most notably, both DDS spin structures, the one at the surface of bulk Fe_1+*y*_Te and thin Fe_1+*y*_Te grown on Bi_2_Te_3_, have a strong component in the out-of-plane direction along the *c* axis, of similar strength as the component in the surface plane. Measurement of the in-plane components of the surface spin-structure of bulk Fe_1+*y*_Te showed that the spin-direction also deviates from the *b*_Te_ axis direction. Neutron scattering, which is sensitive to the bulk magnetization, revealed a dominant spin direction of the DDS structure along the *b*_*Te*_ axis direction^22^,^23^,^26^. A tiny component of the magnetization along the *a*- and *c* axes has been mainly attributed to local moments of excess iron atoms, which are located in the van der Waals gap between the FeTe layers^23^. In contrast, our experiments indicate a strong component of the surface magnetization out of the *b*_*Te*_ axis direction, in favour of a reorientation of the ordered magnetic moments at the surface of Fe_1+*y*_Te. The central question is, thus, what drives this surface reorientation of the spin direction by keeping the overall DDS order.

Owing to its layered crystal structure Fe_1+*y*_Te has a quasi-two-dimensional electronic structure with a very small exchange interaction between the Fe atoms from different layers^34^. It has been shown that the relative orientations of the spins within the DDS spin structure are already determined by the three nearest-neighbour exchange interactions of the Fe atoms within the *a*–*b* plane of a given Fe_1+*y*_Te layer, which are on the order of 10 meV (ref. 35). On the other hand, the absolute orientation of the spins within the DDS spin structure is determined by the magnetic anisotropy energy, which is only on the order of 0.5 meV in the bulk layers^28^. Our result of an overall DDS surface spin structure, which is preserved but reoriented with respect to the bulk layers, suggests that the rather strong exchange interactions within the layers are largely unaffected by the effects occurring at the surface, whereas these effects have a qualitative impact only on the weaker magnetic anisotropy energy. The magnetic anisotropy energy usually has two main contributions, the magneto-crystalline contribution and the dipolar stray field contribution. While the former could be affected by a relaxation of the surface layer with respect to the bulk layers, the latter is usually weak for an AFM spin structure as the DDS structure. We therefore propose that a possible lattice relaxation of the topmost Fe_1+*y*_Te layer could be responsible for the observed reorientation.

Our findings may have important implications for the investigations of the related superconducting material Fe_1+*y*_Se_1−*x*_Te_*x*_ using surface-sensitive techniques. The superconducting pairing in Fe-based superconductors is intertwined with the magnetism in the iron *d*-orbitals^36^. Our results show that the spin direction at the surface is very different from the bulk. By strong spin–orbit interaction^37^, this will have a considerable effect on the energetics of the *d*-orbitals and could thereby also change the superconducting properties at the surface with respect to the bulk^38^. Finally, comparable SP-STM studies could offer insight into the origin of the strongly increased *T*_C_ of the monolayer FeSe grown on SrTiO_3_ compared with bulk FeSe^7^,^8^,^9^.

In conclusion, we have shown that SP-STM experiments with Fe-coated W-tips are well suited to investigate correlated electron systems such as Fe_1+*y*_Te with a complex electronic and magnetic structure. However, a complete characterization requires SP-STM experiments performed in three-dimensional vector-field systems offering field-dependent studies with arbitrary field orientation.

## Methods

### Sample and tip preparation

High-quality Fe_1+*y*_Te single crystals were synthesized using the flux method^39^ where the excess Fe ratio *y* was kept as low as possible. The measured composition of the crystals using single crystal X-ray diffraction resulted in *y*≈7%. The samples were cleaved *in situ* at room temperature and measured in ultra-high vacuum (UHV) with a background pressure better than 3 × 10^−10^ mbar. For all SP-STM measurements the bulk samples were moderately annealed at 100 °C after cleaving, which removes the surface excess iron and leads to an atomically flat surface. In addition, ultra-thin Fe_1+*y*_Te films were grown *in situ* on Bi_2_Te_3_ substrates. Single crystals of Bi_2_Te_3_ were synthesized using a Stockbarger method and were well characterized using angle-resolved photoemission spectroscopy (ARPES)^40^. Fe-chalcogenide thin-film preparation was carried out in a UHV system with a base pressure better than 3 × 10^−10^ mbar. The Bi_2_Te_3_ crystals were cleaved *in situ* under UHV conditions and Fe_1+*y*_Te thin films were prepared by depositing 0.5–1 monolayer Fe onto the clean Bi_2_Te_3_ surface at room temperature followed by a 15 min annealing cycle at ∼300 °C. Fe deposited on Bi_2_Te_3_ reacts with the substrate on annealing, most probably via a substitutional process replacing Bi by Fe. This preparation was performed similar to the method described in ref. 41 for FeSe on Bi_2_Se_3_. Here, the main difference in the growth is that the Fe_1+*y*_Te islands do not show the moiré-pattern observed for FeSe on Bi_2_Se_3_ but a smooth atomically flat surface. To prepare spin-sensitive tips, electrochemically etched W-tips were shortly heated to ∼2,000 °C (flash) and afterwards a thin Fe film of ∼10 nm thickness was deposited onto the tip apex by e-beam deposition^14^,^32^.

### Experimental techniques

The STM experiments were performed in two home-built low-temperature UHV-STM systems at the base temperature of 6.5 K^42^,^43^. One of them was used for the data shown in Figs 2 and 4, to apply either out-of-plane magnetic fields up to 2.5 T or in-plane magnetic fields in a given direction up to 1 T^42^. The other was used for the data shown in Fig. 3, to continuously rotate a magnetic field of 1 T in the surface plane^43^. All STM data were recorded in constant-current mode with a fixed sample bias voltage *V*_bias_ and a constant set-point for the tunneling current *I*_t_. The FTs were calculated from the absolute value of the complex two-dimensional fast FT, which is proportional to the power spectral density.

### Data availability

The authors declare that the main data supporting the findings of this study are available within the article and its Supplementary Information files. Extra data are available from the corresponding author upon request.

## Additional information

**How to cite this article:** Hänke, T. *et al*. Reorientation of the diagonal double-stripe spin structure at Fe_1+*y*_Te bulk and thin film surfaces. *Nat. Commun.* **8,** 13939 doi: 10.1038/ncomms13939 (2017).

**Publisher's note**: Springer Nature remains neutral with regard to jurisdictional claims in published maps and institutional affiliations.

## Supplementary Material

Supplementary Information Supplementary Figures 1-4
