# Supplementary material for: Reorientation of the diagonal double-stripe spin structure at Fe1+yTe bulk and thin-film surfaces
Source: Nat Commun. 2017 Jan 6;8:13939. doi: 10.1038/ncomms13939 (PMC5227097; doi:10.1038/ncomms13939)
Supplement: Supplementary Information — Supplementary Figures 1-4 [file ncomms13939-s1.pdf]

Supplementary Figures:

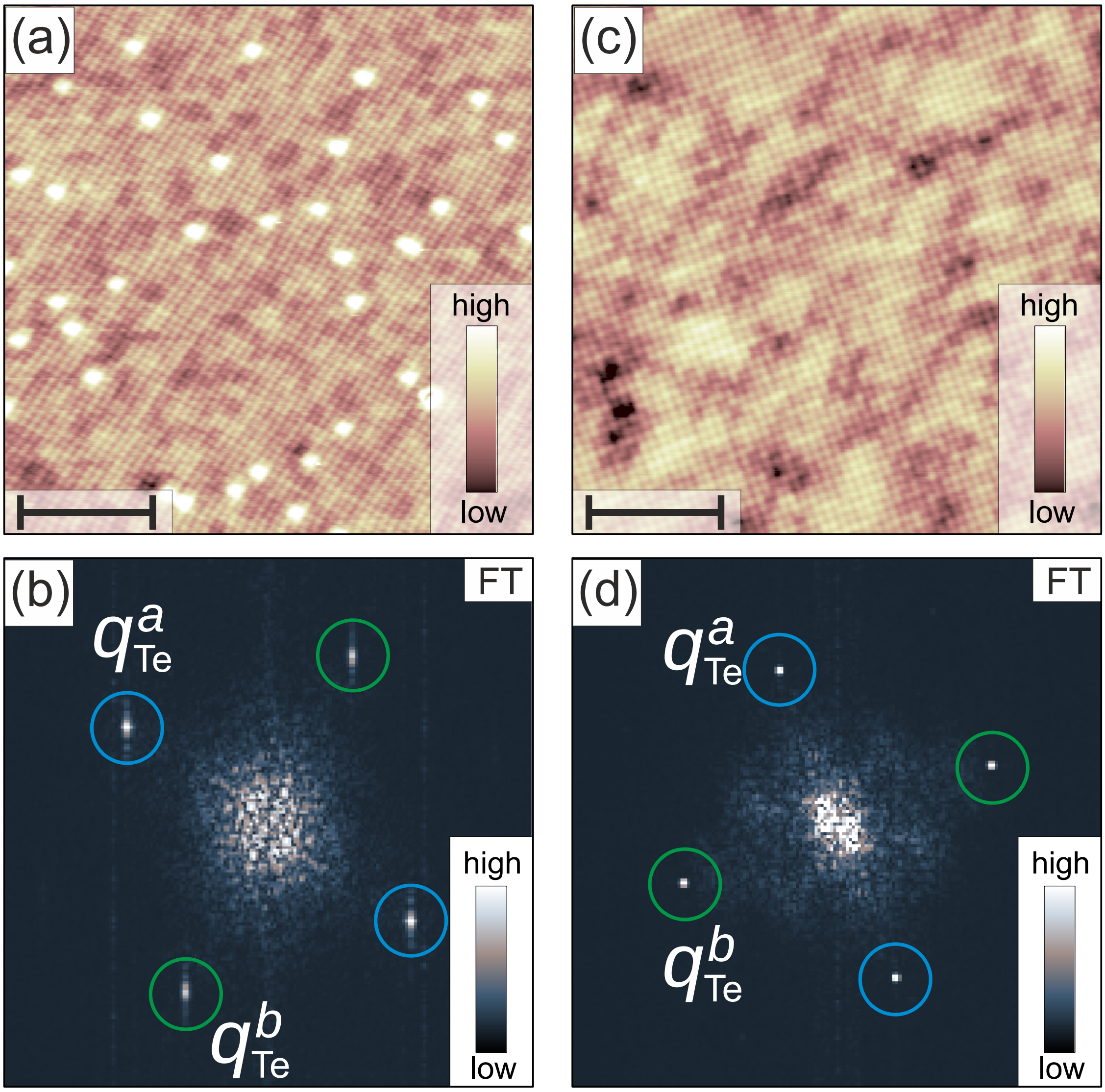

Supplementary Figure 1 | Non-spin-polarized STM images and corresponding FTs. (a) STM image of bulk $Fe_{1+y}Te$ taken with a tungsten tip (V_{bias} = -20 mV, I_t = 390 pA, T = 30.5 K, B = 0 T, 4.8 nm scale bar). (b) FT of (a). (c) STM image of thin $Fe_{1+y}Te$ film grown on Bi_2Te_3 taken with a platinum-iridium tip (V_{bias} = 50 mV, I_t = 300 pA, T = 1.1 K, B = 0.4 T, 5 nm scale bar). (d) FT of (c). In both cases, bulk and thin film, the images reveal the atomic corrugation of the lattice of surface Te atoms without indication of the additional long range superstructure of $2a_{Te}$ periodicity. The latter is only present for spin-polarized tips with a matching orientation of the spin-polarization.

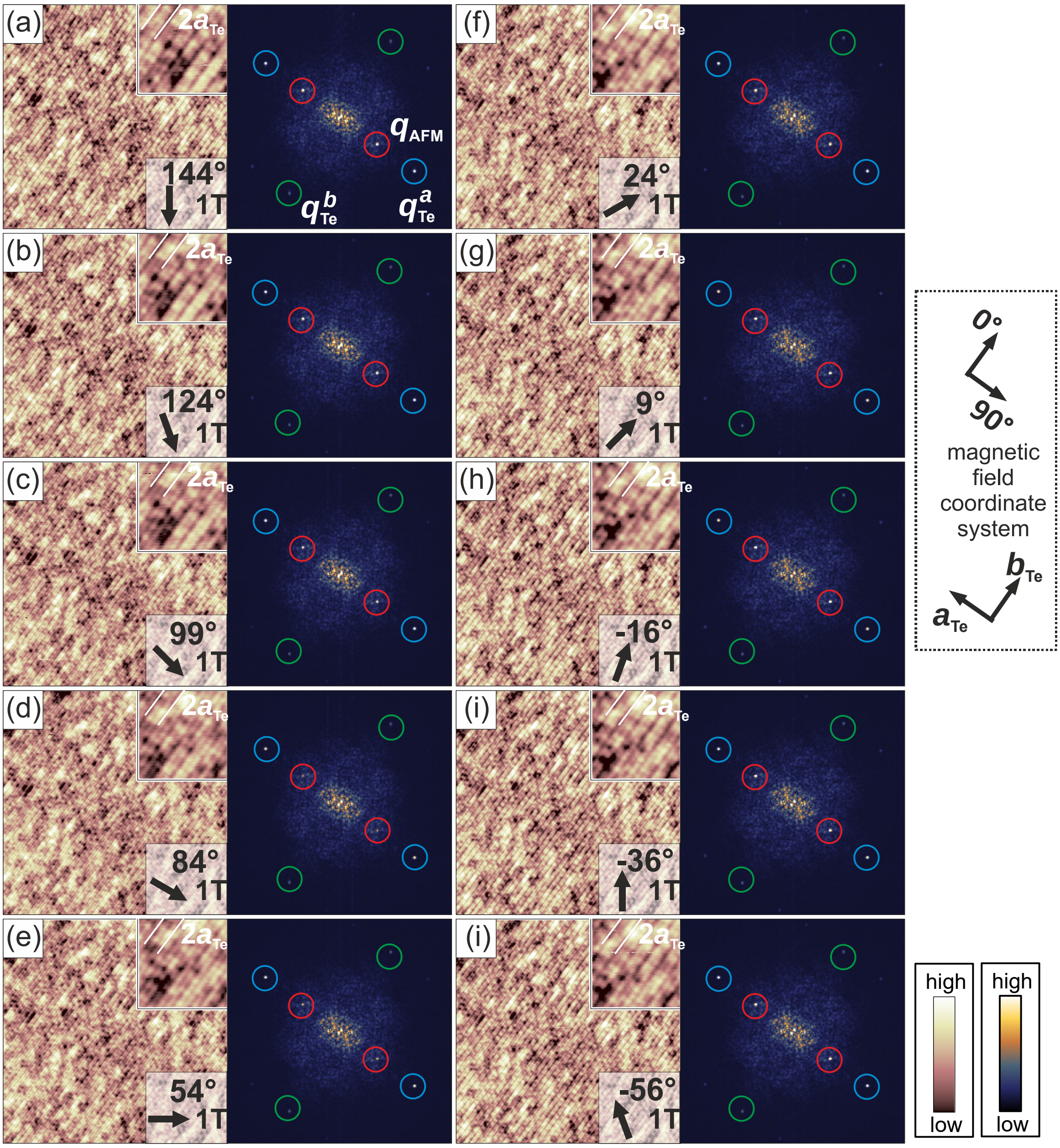

Supplementary Figure 2 | In-plane spin contrast at the surface of bulk $Fe_{1+y}Te$. (a)-(i) show the spin-resolved constant-current images (V_{bias} = +50 mV, I_t = 320 pA, (32.5×32.5) nm^2) of the same field of view for external magnetic fields of 1 Tesla applied in different in-plane directions (left panels) and the corresponding Fourier transforms (FT) (right panels). The direction of the external magnetic field is indicated by the black arrows. The peaks $q_{\mathrm{Te}}{}^{a}$, $q_{\mathrm{Te}}{}^{b}$ and q_{AFM} in the FTs are marked with blue, green and red circles, respectively. (insets) Magnified images showing the atomic lattice of the Te- terminated surface and the $2a_{\mathrm{Te}}$ periodic superstructure. The data of this figure has been used for Fig.3(j) of the main manuscript.

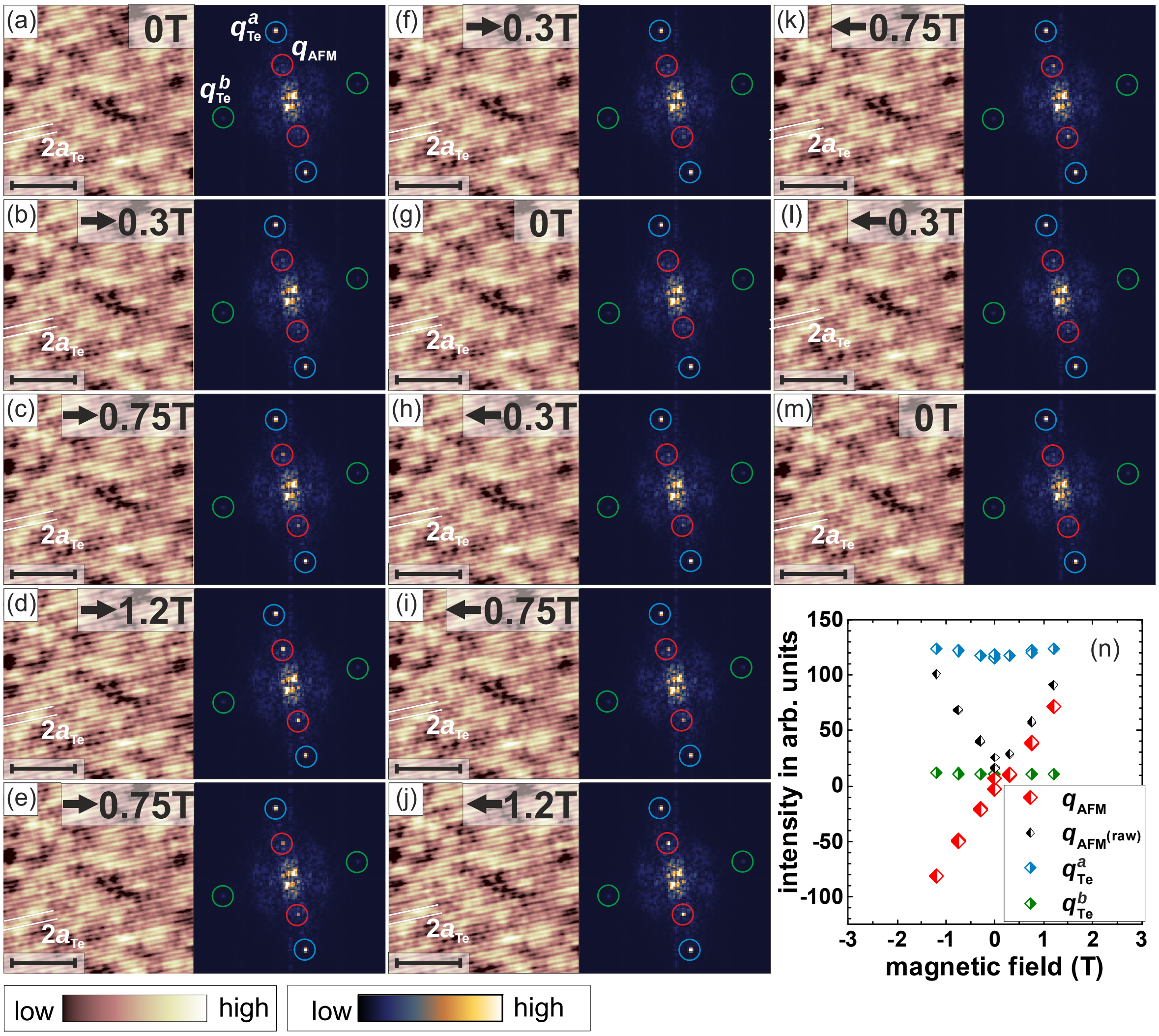

Supplementary Figure 3 | In-plane spin contrast at the surface of thin $Fe_{1+y}Te$ films grown on Bi_2Te_3. (a)-(m) show the spin-resolved constant-current images (V_{bias} = +33 mV, I_t = 4.1 nA, with 3.8 nm scale bars) of the same field of view for external magnetic fields of different amplitudes applied in the in-plane direction (left panels) and the corresponding Fourier transforms (FT) (right panels). The direction and the amplitude of the external magnetic field are indicated by the black arrows and the values in the insets. The peaks $q_{Te}{}^{a}$, $q_{Te}{}^{b}$ and q_{AFM} in the FTs are marked with blue, green and red circles, respectively. The magnetic field dependence of the intensities of $q_{Te}{}^{a}$, $q_{Te}{}^{b}$ and q_{AFM} in the FTs is plotted in (n), where the color coding of the corresponding symbols match the circular markings in the FTs. For q_{AFM} the extracted raw intensities are plotted with black symbols. It is visible, that the intensity of q_{AFM} is getting larger for both field directions upon increasing the magnetic field amplitude. Since a phase shift of the $2a_{Te}$ superstructure by one lattice constant is observed for opposite field directions and the plotted absolute value of the FT contains no phase information, the amplitude for negative field directions was multiplied by -1 in order to transform the FT intensities into values of relative orientation of the spin-directions (red symbols). Furthermore, the background intensity at q_{AFM} at 0 T was subtracted for all red data points of q_{AFM}. The data of this figure has been used for Fig.4(h) of the main manuscript.

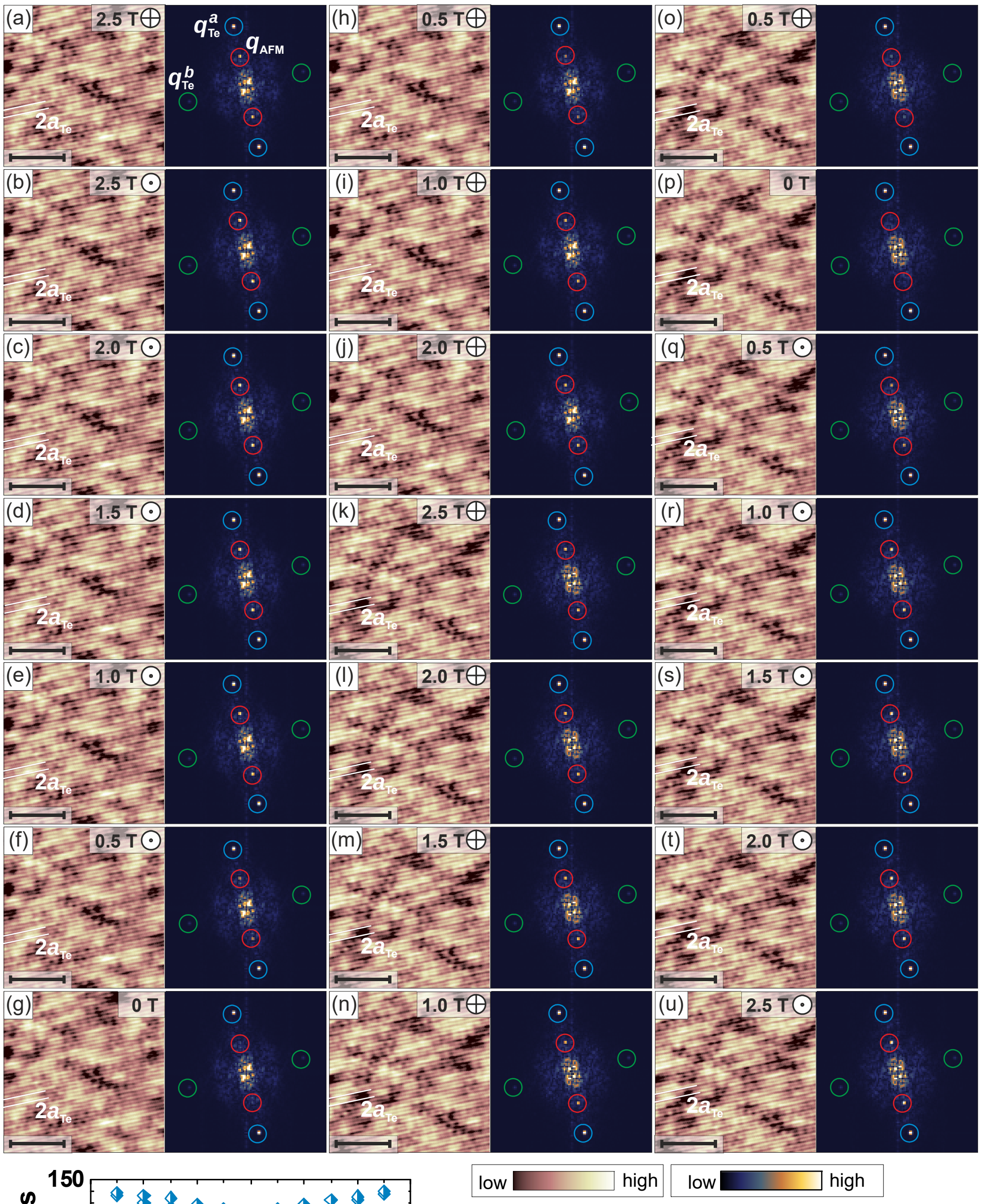

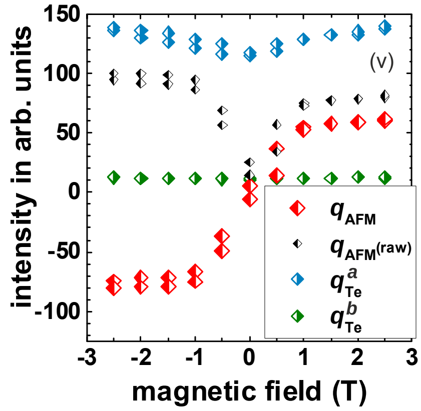

Supplementary Figure 4 | Out-of-plane spin contrast at the surface of thin $Fe_{1+y}Te$ films grown on Bi_2Te_3. (a)-(u) show the spin-resolved constant-current images (V_{bias} = +33 mV, I_t = 4.1 nA, with 3.8 nm scale bars) on the same sample area for external magnetic fields of different amplitudes applied in the out-of-plane direction (left panels) and the corresponding Fourier transforms (FT) (right panels). The direction and the amplitude of the external magnetic field are indicated by the black arrow and the value in the insets. The peaks $q_{Te}{}^{a}$, $q_{Te}{}^{b}$ and q_{AFM} in the FTs are marked with blue, green and red circles, respectively. The magnetic field dependence of the intensities of $q_{Te}{}^{a}$, $q_{Te}{}^{b}$ and q_{AFM} in the FTs is plotted in (v), where the color coding of the corresponding symbols match the circular markings in the FTs. For q_{AFM} the extracted raw intensities are plotted with black symbols. Similar to Fig. S3, the intensity of q_{AFM} for negative field was multiplied by -1 in order to transform the FT intensities into values of relative orientation of the spin-directions (red symbols). Furthermore, the background intensity at q_{AFM} at 0 T was subtracted for all red data points of q_{AFM}. The data of this figure has been used for Fig. 4(d) of the main manuscript.
